# Mesenchymal stromal cell derived extracellular vesicles as a therapeutic tool: immune regulation, MSC priming, and applications to SLE

**DOI:** 10.3389/fimmu.2024.1355845

**Published:** 2024-02-08

**Authors:** Christophe Wong, Ivana Stoilova, Florence Gazeau, Jean-Philippe Herbeuval, Thibaut Fourniols

**Affiliations:** ^1^ EVerZom, Paris, France; ^2^ Centre National de la Recherche Scientifique (CNRS) Unité Mixte de Recherche (UMR) 8601, Université Paris Cité, Paris, France; ^3^ Chemistry and Biology, Modeling and Immunology for Therapy (CBMIT), Université Paris Cité, Paris, France; ^4^ Matière et Systèmes Complexes (MSC) UMR CNRS 7057, Université Paris Cité, Paris, France

**Keywords:** extracellular vesicles, secretome, immune regulation, mesenchymal stromal cell, priming, systemic lupus erythematosus

## Abstract

Systemic lupus erythematosus (SLE) is a chronic autoimmune disease characterized by a dysfunction of the immune system. Mesenchymal stromal cell (MSCs) derived extracellular vesicles (EVs) are nanometer-sized particles carrying a diverse range of bioactive molecules, such as proteins, miRNAs, and lipids. Despite the methodological disparities, recent works on MSC-EVs have highlighted their broad immunosuppressive effect, thus driving forwards the potential of MSC-EVs in the treatment of chronic diseases. Nonetheless, their mechanism of action is still unclear, and better understanding is needed for clinical application. Therefore, we describe in this review the diverse range of bioactive molecules mediating their immunomodulatory effect, the techniques and possibilities for enhancing their immune activity, and finally the potential application to SLE.

## 1 Introduction

Systemic lupus erythematosus (SLE) is a chronic autoimmune disease, associated with multiorgan damage and variable clinical manifestations (1). SLE is a multifactorial disorder, in which the phenotype is modulated by a combination of genetic, epigenetic, environmental, hormonal and immunoregulatory factors. SLE is characterized by a dysfunction of the immune system, notably a presence of autoreactive T cells and hyperactive B cells, leading to a loss of tolerance, production of autoantibodies against self-antigens formation and deposition of immune complexes, as well as a sustained systemic inflammation. SLE is classified as interferonopathy, since type I interferons (IFN-I) play a crucial role in the development of the disease (2). The chronic production of IFN-I, especially IFN-α, is a key characteristic of SLE and contributes to the autoimmune process (3). IFN-I stimulates the activation of interferon producing cells, such as plasmacytoid dendritic cells (pDCs), which are responsible for elevated levels of IFN-α in blood plasma and organs (4). The excessive signaling of IFN-I results in the increased expression of various pro-inflammatory cytokines, chemokines, and markers of immune cell activation. This, in turn, contributes to the dysregulation of the immune system and the generation of autoantibodies. Additionally, prolonged exposure to IFN-I can boost the activation and survival of autoreactive B cells and encourage the differentiation of T cells into pro-inflammatory subsets, sustaining the autoimmune response (5). SLE manifests through periods of flares and remissions, with symptoms showing considerable variation among individuals. Treatment objectives revolve around managing symptoms, mitigating inflammation, and safeguarding against organ damage. Due to its clinical heterogeneity and complex pathogenesis, SLE remains hard to diagnose and the available treatments show limited efficiency.

Long considered as platelet debris, EVs have gained critical interest from the scientific community in the recent years (6). EVs are released by all cellular organisms and can be defined by their heterogeneity. Indeed, apart from their structural definition, a cell secreted particle enclosed by a lipidic bilayer, EVs differ from their sources, biogenesis, biophysical, biochemical characteristics and functional activity (7). A standard classification can be established based on the biogenesis of EVs: exosomes are generally small sized extracellular vesicles, from endosomal origin, released by multivesicular bodies (MVBs). Ectosomes can be defined as extracellular vesicles stemming from the plasma membrane. EV biogenesis pathways and mechanisms of interaction with target cells have been extensively reviewed (8, 9). EVs are heterogenous in size and have a diameter varying in the range of the nanometer. However, EVs are not the only nanometer-sized particles secreted by cells and there is still no specific markers allowing an efficient separation of EVs. Thus, guidelines have been set by the international community regarding ways of purifying and characterizing EVs. Transmembranal proteins such as tetraspanins, cytosolic proteins such as TSG101, and other cell-dependent proteins have been used to demonstrate the EV nature and to some extent the degree of purity of an EV preparation (10). EVs have been linked with a variety of cellular functions, and can affect other cells with their surface proteins or the cargo encapsulated by EVs. EVs carry diverse proteins, nucleic acids and lipids from their parent cell, which can in turn be delivered to the recipient cell. EVs have a fundamental role in the immune system and in immune-related diseases, highlighting their potential either as a new biomarker or a therapeutic tool (7).

In the field of mesenchymal stromal cells (MSCs), EVs have emerged as a potential avenue for cell-free therapy. MSCs have been used for their immunosuppressive capabilities, with utilization in clinical trials and availability on the market. Nonetheless, the use of MSCs can carry some drawbacks and EVs have been looked into as a cell-free alternative. More specifically, as EVs carry the same biological molecules as their parent cells, the effect of MSCs is partially mediated by them with paracrine actions. EVs have shown to have the same immunomodulatory and regenerative potential than their parent cells, and have thus become a promising alternative to MSCs themselves for therapies (11, 12). More broadly, the secretome embraces not only the previously mentioned EVs but also a variety of proteins, lipids, and nucleic acids. These components may be either inside or adsorbed to EVs, or freely present in the secretome (13). This added complexity of which fractions holds the therapeutical activity and the issues of standardization regarding means of production, concentration, and characterization in the field of EVs make any conclusion towards the effect of MSC derived EVs or MSC derived secretome a complex equation.

## 2 Multiparametric influence on the immunomodulatory potential of MSC-EVs

In order to understand which fractions hold the immunomodulatory potential, the next part focuses on having a critical look over the many parameters influencing MSC-EV immune activity. Some scientific papers use the term exosomes to describe their fractions, while they could be more accurately defined as EVs or even secretome. Various downstream processes such as size exclusion chromatography (SEC), polyethylene glycol (PEG) and ultracentrifugation broadly used by the scientific community separate selectively the content of the secretome (14), and alter the composition of the protein corona of EVs (15). The protein corona is a set of proteins and other molecules adsorbed to the EVs. These proteins can be bioactive and mediate part of the immunomodulatory potential (16, 17). All of this heterogeneity of methods brings at the same time strength to the immunomodulatory response of EVs as a global effect, but brings hardship as to determine what is driving this specific effect. A number of studies showed the difference of potency between the secretome, the EV free fraction, and the fractions containing EVs. Papait et al. showed that amniotic MSCs derived conditioned media (e.g secretome) and the EV free fraction (meaning the supernatant after ultracentrifugation) maintained their immunomodulatory potential by inhibition of T cell, promotion of Tregs, shifted monocytes towards M2 instead of M1, but also reduction of the maturation of dendritic cells. The EV fraction collected after ultracentrifugation had no effect even though these EVs were uptaken (18). Another study has found no effect of the three fractions on the inhibition of T cell proliferation (19). These differences in results might be from methodological differences, specifically in the dose parameter (e.g. protein amount in ug or particles concentration measured by Nanoparticles Tracking Analysis (NTA)), cell sources of EVs, or downstream processes. A recent study has shown that the immunomodulatory potential of MSCs is independent of EVs, which runs counter to most results in the literature using EV-enriched fractions (20). González-Cubero et al. showed that EVs and soluble fractions from conditioned media promote an *in vitro* anti-inflammatory modulation in intervertebral disc degeneration in a “highly synergistic way”, thus highlighting that the use of the whole secretome rather than isolated EVs might be more beneficial for therapy (21). While it is still hard to understand what exactly mediates the immunomodulatory potential, the effect might come from a synergistic combination of both the soluble factors from the secretome and the EVs.

Other factors have also been shown to have an impact on the immunomodulatory potential of EVs or secretome. Indeed, the donor of primary cells, the source of MSCs, the passage doubling number, and the production method (which will be addressed later on) can influence the secretome content and thus the potency of EVs (22–25). Immortalized MSCs might be a solution for reproducible batches of secretome/EVs for therapeutic use (26).

The characterization of the secretome of MSCs shows the presence of both pro and anti-inflammatory molecules. While the secretome of MSC-EVs has shown to have high levels of IL-6 and IL-8 (27), a recent study showed that the conditioned media derived from umbilical cord (UC)-MSC promoted anti-inflammatory macrophages polarization. This despite a mostly pro-inflammatory profile of cytokines, though the authors have only investigated surface markers of M2 macrophages (28). MSC-EVs contain nucleic acids, which may bind to TLR7 and 9 as foreign nucleic acids and trigger a pro-inflammatory cascade. However, this review hasn’t found any reports of inflammation induced by MSC-EV-associated DNA (29).

The methods to assess EV immune potency are also critical. The dose of EV is differently calculated, either with particles concentration, protein concentration, or even EVs per receptor cells, which may result in some disparities in results (30). Some studies tend to isolate a specific cell type for their *in vitro* potency, though immune cells do not uptake homogenously EVs. Monocytes seem to uptake the highest proportion of MSC-EVs (18, 31, 32). Nonetheless, even though EVs are not uptaken by lymphocytes, studies on MSC-EVs and on EV-free fractions added to PBMCs have shown an immunosuppressive effect on T cells, thus independent of the uptake (18, 33, 34). Others have shown that MSC-EVs failed to suppress lymphocyte proliferation (35). The immunomodulatory effect of MSCs on B cells is independent of secreted EVs (36). This reinforces that the global immunomodulatory effect might not be entirely mediated by EVs, but also through soluble factors of the secretome. The effect of EVs on T cells could also be mediated in an indirect way, through the actions on EV-uptaking immune cells (37, 38).

Regarding the immunoregulatory effect of MSC-EVs on immune cells, some reviews have already discussed it thoroughly, either as a comprehensive overview (39), or more precisely in the case of SLE (40). Thus, in the next part, this review will focus on the immunomodulatory factors in the secretome of MSCs.

## 3 The immunomodulatory bioactive molecules of MSC-derived secretome and EVs

**Figure 1:**
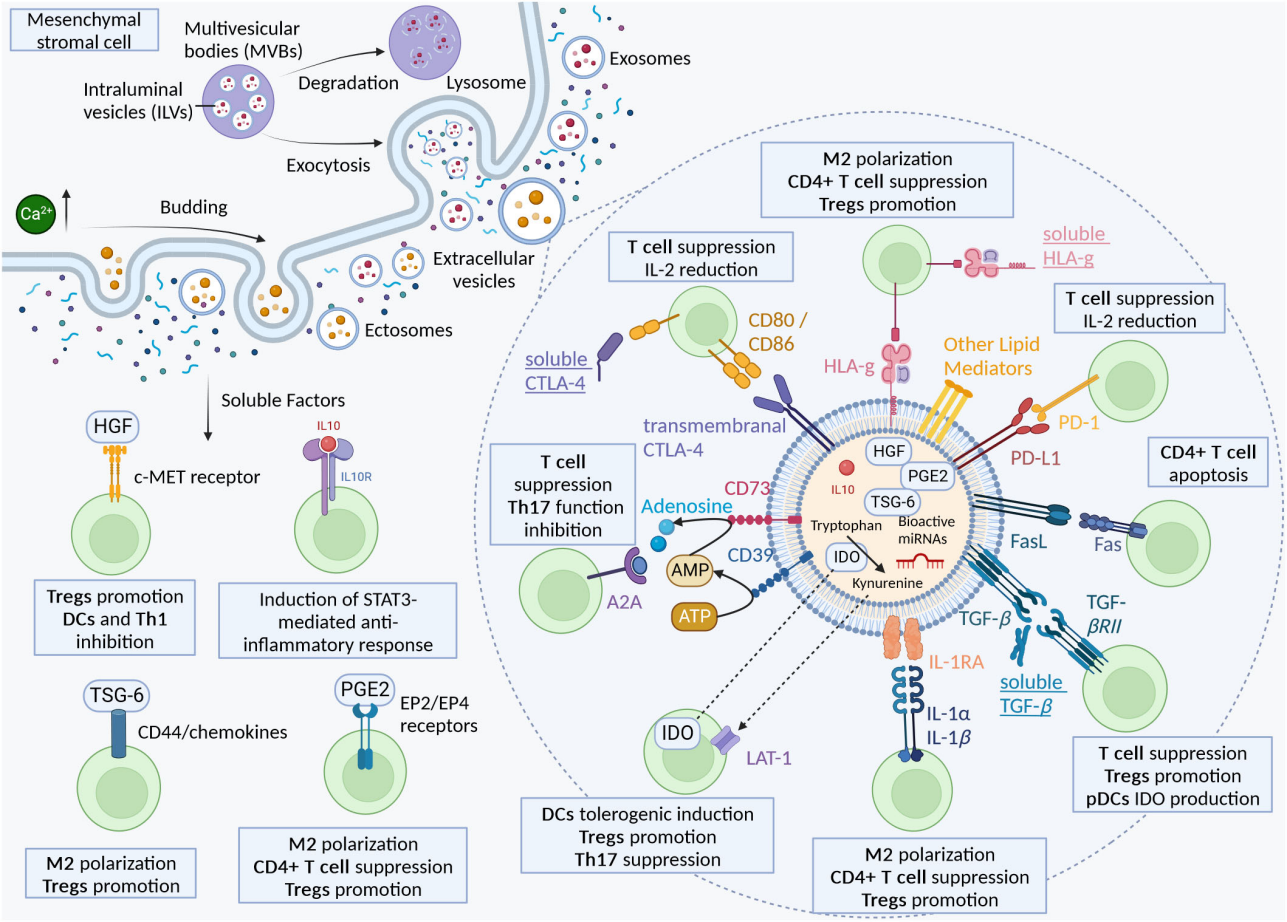
The immunomodulatory bioactive molecules of MSC-derived secretome and EVs.

The immunomodulatory potential of MSC-EVs could be driven by a broad range of bioactive molecules, including proteins, nucleic acids, and lipids. Many evidences suggest the importance of specific bioactive proteins mediating the immunomodulatory effect of MSC-EVs. This review focuses on the supposed localization of the bioactive molecules, though it is important to remember that soluble proteins secreted by MSCs are also probably carried by EVs. The lack of studies comparing EV-free conditioned media and purified EV fractions makes the localization of all bioactive molecules a hard task. (**Figure 1**) is a proposition of the immunomodulatory bioactive molecules of MSC-derived secretome and EVs.

### 3.1 Soluble proteins secreted by MSCs

The immune regulation of MSCs can be mediated by soluble proteins secreted by MSCs.

IL-10 is an anti-inflammatory cytokine secreted by immune cells such as macrophages, dendritic cells, Th and Tregs, amongst others. IL-10 induces a strong immunosuppressive response in immune cells, targeting specific genes, cytokines and chemokines production. An in-depth focus on IL-10 and its effects has already been reviewed (41). IL-10 is also secreted by MSCs and mediates part of MSC-EVs immunomodulatory activity. Jiang et al. showed cardioprotective effects of MSC-EVs using *in vivo* models of pigs, which were “largely blunted” after IL-10 knockdown (IL10KD). Using *in vitro* models, IL10KD MSC-EVs achieved less inhibition of T-cell proliferation than control MSC-EVs (42). These results are in accordance with Eirin et al. observation on their model of kidney inflammation, showing an IL-10 dependent immunomodulation of MSC-EVs (43). MSC-EVs derived from MSC overexpressing IL-10 showed higher concentrations of IL-10, enhanced the suppressive effect of these EVs on Th1 and Th17 and upregulated Tregs *in vitro* (44). IL-10 thus mediates the immunosuppressive effect of MSCs.

Hepatocyte growth factor (HGF) is secreted by MSCs, which can mediate immunosuppressive effects. The effect of HGF in the scope of MSCs has already been described (45, 46). Chen et al. showed that knockdown of HGF secretion by MSCs abrogate the suppression of T cell proliferation, and monocytes cultured with HGF alone or MSCs can secrete high levels of IL-10 through ERK1/2 pathway (47). In the case of MSC-EVs, treatment with MSC derived “microvesicles” reduced IL-6 production and increased IL-10 production in the conditioned media of endothelial cells, which was reverted after knockdown of HGF in MSCs. Notably, MSC-derived conditioned media had a higher regulative activity than microvesicles (48).

Tumor necrosis factor-inducible gene 6 protein (TSG-6) is a protein implicated in the immunomodulatory effects of MSCs. TSG-6 is a multifunctional protein with anti-inflammatory properties, and can interact with a broad variety of ligands such as chemokines. TSG-6 is overexpressed in a pro-inflammatory environment (49). Chaubey et al. showed the presence of TSG-6 in UC-MSC-EVs was linked to the therapeutic efficiency in their model of mouse lung disease (50). TSG-6 in canine MSC-EVs played a key role in the downregulation of pro-inflammatory cytokines, the polarization of M1 to M2 and the increase of Tregs in the colon (51). Human bone marrow(BM)-MSC derived EVs containing TSG-6 decreased pro-inflammatory cytokines in scar tissues, inhibited collagen deposition, thus suppressing scar formation. This effect was enhanced using BM-MSC modified to overexpress TSG-6, and reverted to the normal after knockdown, showing a TSG-6 dependent effect of MSC-EVs (52). Lu et et al. showed that AD-MSC derived EVs had therapeutic effects in their model of spinal cord ischemia reperfusion injury by transmitting TSG-6 (53). Other studies have shown the importance of TSG-6 in MSC conditioned media for immunomodulatory activity (54, 55).

### 3.2 Proteins adsorbed to the corona & membrane proteins

IL-1 receptor antagonist (IL-1RA) has also been found in the secretome of MSCs. IL-1α and IL-1β, potent inflammatory cytokines can bind to IL-1R, eliciting a MyD88-dependent inflammatory cascade. On the other hand, IL-1RA can bind without triggering any downstream signaling, therefore acting as a potent antagonist of IL-1a and IL-1β and shutting down immune responses. An in-depth report of the actions of MSC-derived IL-1RA can be found (56). Kou et al. showed that IL-1RA was found in the supernatant of cultured mouse MSCs after centrifugation at 3,000 g and 20,000 g, and not in the pelleted fraction representing the bigger EVs, but was found in was in the EV pelleted fraction they call “small EVs” after ultracentrifugation at 120 000g. Using various methods of microscopy, they showed that IL-1RA was carried by EVs on their surface. They subsequently demonstrated that IL-1RA associated EV release was controlled by Fas through binding with Fap-1 and Cav-1 and upregulated when the MSCs were treated with TNF-α (57).

Transforming Growth Factor Beta (TGF-β) is a cytokine carried by MSC-EVs (58). TGF-β can bind on its receptor TGF-βRII, which triggers a downstream cascade targeting a variety of growth factors and inflammatory cytokines. TGF-β1 can be found either bounded to the plasma membrane, or in soluble form (59). TGF-β has a pleiotropic function on the regulation of immune cells. TGF-β suppresses T cells while promoting Tregs, regulates B cell activation, promotes expression of IDO in pDCs, inhibits DC function, amongst other actions (60, 61). TGF-β has been studied in MSC-EVs regarding its immunosuppressive activity. Alvarez et al. showed that TGF-β1 was primarily present in MSC-EV fraction, compared to EV free supernatant and that the immunomodulatory activity of MSC-EVs on CD4+ T cells is partially mediated by TGF-β1 (62). This same conclusion has been advanced by another group in a canine model (34). Kim et al. showed that amongst other molecules, TGF-β had a significant influence on the immunomodulatory properties of MSC-EVs in their model of cornea. Silencing of TGF-β1 resulted in the loss of MSC-EV suppressive effects, while overexpression resulted in more effective EVs in the suppression of T-cell receptor IL-2 and IFN-γ secretion in activated splenocytes (25). Song et al. used MSC-EVs produced in 3D with exogenous TGF-β3, which resulted in higher levels of TGF-β1 compared to non-treated 3D MSC-EVs, and higher immunomodulatory activity of treated EVs (63).

CD73 is one of the three conventional surface markers to identify MSCs (64). But it is also one of the enzyme of purinergic signaling, responsible for transforming Adenosine monophosphate (AMP) into Adenosine (Ado), a nucleoside known for its immunosuppressive role on T cells and Th17 cells (65). Indeed, CD73+ engineered UC-MSC derived EVs reduced concentration of ATP while increasing the levels of adenosine compared to non-engineered EVs. These engineered EVs improved the functional recovery after spinal cord injury, improving the polarization from M1 to M2 phenotype, but also downregulated more the pro-inflammatory cytokines after spinal cord injury compared to the native EVs, while it also upregulated more the anti-inflammatory cytokines such as IL-10 (66). Another study has shown that conditioning of MSCs with pro-inflammatory cytokines promoted the expression of CD73 in EVs, and that these EVs reprogram macrophages from M1 to M2 phenotype (67). Although MSCs and hence their EVs express CD39 (at a low level) and CD73, a study has shown that efficient adenosine production from ATP requires cooperation with activated T-cells expressing CD39 (68). The co-culture in the previous study significantly increased the expression of CD39 in MSCs and of CD73 in T-cells, supporting the previous findings of Saldanha-Araujo et al. (69). In the case of SLE, patients show a silenced activity of CD73 and CD38 in B cells, resulting in decrease of production of anti-inflammatory adenosine (70). MSC-EVs may thus constitute a potential therapeutic approach for the treatment of SLE due to their high expression of CD73. Other methods of overexpressing this enzyme could be also used to further improve the treatment.

#### 3.2.1 Immune checkpoints

Cytotoxic T lymphocyte antigen 4 (CTLA-4) is an important regulator of T cell activation (71). It can bind to CD80 and CD86, resulting in an inhibition response rather than a stimulatory one by CD28. Furthermore, CTLA-4 can also bind to DCs, resulting in a downregulation of CD80 and CD86 (72). CTLA-4 expressed on Tregs stabilizes the interaction with T cells allowing for the Treg mediated suppression of T cell (73). CTLA-4 has been shown to be expressed by MSCs under different isoforms. CTLA-4 can be found as a transmembrane protein or can also be secreted. Secreted CTLA-4 by BM-MSCs has been significantly increased under hypoxic conditions. The authors have also shown a CTLA-4 mediated inhibitory effect of the secretion of TNF-α induced by PHA of PBMCs (74). As far as we know, there are no studies directly showing the presence of CTLA-4 in the membrane of MSC-EVs. As CTLA-4 is expressed in MSCs, their derived EVs could potentially carry it.

On the contrary, PD-L1 has been found on the surface of MSC-EVs. PD-L1 binds to the receptor programmed death-1 (PD-1), expressed on T cell surface, leading to an inhibition of their activation (75, 76). Wu et al. have shown that PD-L1 overexpressing MSC-EVs have enhanced therapeutic activity compared to native EVs in a model of LPS-induced pneumonia in mice (77). Other teams also used PD-L1 overexpressing MSC-EVs, showing an increased therapeutic activity in their *in vitro* and *in vivo* models. Notably, the use of anti-PD-L1 antibody reverted the effect to the level of wild type MSC-EVs, suggesting a PD-L1/PD-1 dependent immunosuppression (76–79). Pro-inflammatory and hypoxia MSC conditioning resulted in higher levels of IDO and PD-L1 in EVs, resulting in higher immunomodulatory activity (80). In regard to SLE, CD4+/CD25+/Foxp3+ Tregs from SLE patients expressed significantly lower amounts of PD-L1 compared to healthy patients (81).

HLA-G is a non-classic major histocompatibility complex (MHC) molecule which can mediate immunosuppression. HLA-G can bind to receptors expressed in immune cells, such as CD158d, CD85j, CD85d, CD8 and CD160, and can polarize macrophages towards an M2 phenotype, inhibits the proliferation of T cells, induces Tregs, inhibits the maturation of DCs amongst other actions. There are seven different isoforms, with four membrane bound (HLA-G1, G2, G3, and G4), and three soluble isoforms (HLA-G5, G6, and G7) (82–85). A limited number of studies have examined the impact of human leukocyte antigen G (HLA-G) in MSC-EVs. Selmani et al. showed that HLA-G5 can be found in the secretome of BM-MSCs. BM-MSCs secreted HLA-G5 in an IL-10 dependent manner. Interestingly, they noted a decrease of HLA-G expression over passages, but HLA-G5 content in supernatants was not affected. They further demonstrated that HLA-G5 is necessary for the suppression of allogenic T-cell and the expansion of Tregs, and the inhibition of “NK-cell mediated cytolysis and interferon-y secretion” (86). HLA-G has been found in “high levels” of MSC-EVs of four bone marrow donors, purified by PEGylation and ultracentrifugation (87). This could indicate that HLA-G can indeed be found in the secretome, but also linked with MSC-EVs. In the same vein, HLA-G, in both its membrane and soluble isoforms, has also been identified in UC-MSCs, without specifying their EVs (88).

#### 3.2.2 Apoptose inducing ligands

FasL or CD178 or CD95 Ligand induces apoptotic death upon binding with its receptor Fas. Interestingly, FasL induces cell death only in its membrane-bound form (mFasL) while its soluble form (sFasL) binds to Fas without induction of the proapoptotic signaling pathway, thus competing with its membrane-bound form (89). sFasL is found after cleavage of mFasL by metalloproteinases (MMPs). sFasL can trigger inflammatory pathways such as NF-κB (90). As a matter of fact, MMPs are found in the secretome of MSCs after priming with IL-1β (91), and might cleave FasL into sFasL at the surface of MSCs and their EVs, which might in turn compete with FasL. It is not known in the detail in the case of MSCs and EVs how this dynamic between pro-inflammatory sFasL, MMPs and pro-apoptotic mFasL plays out regarding the immunomodulatory potential of MSC-EVs. Some studies have shown a FasL dependent apoptotic effect using MSCs, but little has been done with MSC-EVs. Vacaru et al. used modified MSCs overexpressing FasL which showed improved death induction in CD4+ and CD8+ T cells, but has not looked into the effect of EVs (92). Akiyama et al. showed that bone marrow MSCs induced T-cell apototosis via Fas/FasL pathway. Recruitment of T cells was induced by secretion of Fas-regulated monocyte chemotactic protein 1 (MCP-1) (93). Wang et al. induced apoptotic EVs in mouse MSCs, and showed that these EVs used FasL to induce apoptosis in Multiple myeloma cells *in vivo* in mice (94).

### 3.3 EV cargos

Indoleamine 2,3-dioxygenase (IDO), is an immunosuppressive enzyme leading to the degradation of tryptophan to kynurenine and other metabolites, resulting in stopping T-cell proliferation, induction and activation of regulatory T cells (95). Kynurenine produced by DCs can be taken up by T cells through the large neutral amino acid transporter (LAT-1), and induces FoxP3 expression, resulting in Tregs differentiation. Kynurenine also inhibits the expression of retinoic acid receptor-related orphan receptor-γt (ROR-γt), thus suppressing the differentiation of Th17 cells. Kynurenine can also induce tolerogenic phenotype in DCs (96). IDO activation in plasmacytoid dendritic cells (pDC) induce immune tolerance and inhibition of IFN-I production (97, 98). As SLE is characterized by an enhanced IFN-I production by pDC, IDO could be a key immune modulator of MSC-EVs in SLE treatment. In MSCs, IDO is present in MSC-EVs and in cells, but only after IFN-y pre conditioning. Increased levels of IDO expression result in higher levels of Kynurenine, both in the cell and in the EVs. EVs unprimed with IFN-γ showed no immunomodulatory properties, while primed EVs suppressed T-cell and induced Treg cells (99). MSC-EVs from overexpressing IDO MSCs have shown to activate M2 polarization in an IDO dependent manner (100). Another study showed an effect of unprimed MSC-EVs on inhibition of PBMC proliferation, but no statistically significant increase of Tregs proportion. Treatment with MSC-EVs primed with IFN-γ and TGF-β showed a higher increase in the proportion of Tregs compared to conditions primed with only one molecule, or unprimed. This increased immunomodulatory activity was linked with a higher concentration of IDO and IL-10 (101). However, Serejo et al. showed that while IFN-y pre-treatment of MSCs did increase the expression of IDO in both cells and EVs, it did not result in increased T-cell suppression of proliferation (102). Another study found that conditioned media from primed cells successfully suppressed T cell proliferation, while primed MSC-EVs, and unprimed CM and EVs had no effect (103). The authors hypothesize that the absence of immunomodulatory effect could be due to a difference in experimental methodology. These studies show that IFN-γ priming is needed for IDO expression, which could mediate the immunosuppressive effect of MSC-EVs.

Regarding the iNOS-NO axis, the immunosuppressive activity of MSCs from human, monkey and pig is mostly mediated through IDO rather than iNOS, whereas MSCs derived from rat, hamster and rabbit mostly use iNOS (104). For that reason, we are redirecting to another review which has already described in-depth the role of iNOS in the immunomodulatory potential of MSCs and their EVs (105).

#### 3.3.1 miRNAs

The immunomodulatory potential of MSC-EVs could also be mediated through miRNAs. miRNA are non-coding small nucleic acid of around twenty nucleotides which interfere with mRNA translation. As such, when delivered to the immune cells, miRNAs can influence their function through the inhibition of transcription factors or targeting of specific pathways regarding maturation, activation of the immune cells (106). miRNAs can be transported and delivered to other cells by extracellular vesicles, which make them a potent molecule responsible for the immunomodulatory potential of MSC-EVs. MSC-EVs have been found to be enriched in various miRNAs, with differentially expressed miRNAs depending on the source of the MSC-EVs (107). This should be kept in mind looking at potential immunomodulatory mediated miRNAs action as they could be weakly enriched in a specific source of MSC-EVs.

Kim et al. identified let-7b-5p and miR-21-5p as key microRNAs mediating the immunomodulatory effect of MSC-EVs (25). miR-21-5p, identified as one of the most enriched miRNA in MSC-EVs, targets CCR7 resulting in attenuated DC migration and function (108). miR-146a has been shown by Song et al. to promote M1-M2 transition and plays a protective role in sepsis (109). miR-181c in UC-MSC-EVs repressed inflammation by suppressing TLR4 (110). miR-155 has been shown to significantly reduce the proliferation of activated PBMCs, by targeting miR-221 as a potential inflammation mediator (111). miR-223 in MSC-EVs could also restrain adhesion and migration of T cells (112). A lot of other miRNAs have been identified as a potential mediator of MSC-EVs immunomodulatory effects (113). A few studies have highlighted limitations of current RNA-sequencing methods, which could raise concerns over the reproducibility and comparability of sequencing data across library preparation platforms (114, 115). Nonetheless, Srinivasan et al. indicate that technical variability is smaller than biological variability regarding the use of small RNAs in EVs (116). miRNAs have thus shown to mediate a broad Immunosuppressive effect of MSC-EVs.

#### 3.3.2 Lipid mediators

While the scientific community has been mainly focused on the immunomodulatory impact of miRNAs and various proteins, some bioactive lipids have been coming in light.

Lipid mediators are lipids derived from polyunsaturated fatty acid (PUFAs) who can promote inflammation or resolution. PUFAs can be modified by different types of enzymes into a diversity of bioactive lipids. Among them, specialized pro resolving mediators (SPMs) such as resolvins or maresins are known to be anti-inflammatory and play a key role in the resolution of inflammation. The regulatory effect of SPMs on immune cells has already been described extensively (117, 118). Regulatory lipid mediators have been found in MSC-EVs (119–121), and their concentration can be increased by priming MSCs with PUFAs, or even with pro-inflammatory cytokines (121, 122). Enzymes of the many lipid mediators pathways are carried by MSC and their EVs. Cardiac MSCs and their EVs carry 5-LO and 15-LO, enzymes which play a role in transforming PUFAs in SPMs (123). Thus, these enzymes are able to modify dynamically the concentration in various SPMs inside EVs. PUFA supplementation of MSCs to improve the immunomodulatory potential has already been described in the literature (124, 125). Secretome of primed MSC with different types of PUFA promoted an immunoregulatory phenotype in macrophages (126). While lipid mediators may play a role in the immunomodulatory effect of MSC-EVs, little research has been done about them.

Prostaglandin E2 (PGE2) is a lipid mediator belonging to the prostaglandin family. PGE2 is produced by the enzymes COX-1 and COX-2 from the ω-3 PUFAs arachidonic acid (AA) and can bind on EP receptors expressed on the surface of immune cells (45). EP2 and EP4 receptors, upon binding, upregulates cAMP levels whereas EP3 downregulates it (127). PGE2 binds on EP2 and EP4 receptors and regulates T helper cells (128). PGE2 has shown to have a broad immunomodulatory effect. It promotes an anti-inflammatory phenotype in macrophages, has ambivalent effects on DCs depending on their development stage, suppresses T cell activation and promotes Treg cells (129, 130). PGE2 can also induce neutrophils to produce less pro-inflammatory lipid mediators and increase the production of anti-inflammatory lipid mediators such as Lipoxins (131). In prostate cancer cells, PGE2 has been found either to be carried with EVs but also secreted as a soluble factor (132). PGE2 is expressed in MSCs, carried by their EVs, and the cells can be primed with ω-3 PUFA to express more PGE2 (121). Conditioned media from human MSCs spheroids inhibited pro-inflammatory cytokines and increased the secretion of anti-inflammatory cytokines in LPS stimulated macrophages, in a PGE2 dependent manner, mediated by binding on the EP4 receptor (133). MSC-EVs from pluripotent stem cells isolated by anion-exchange chromatography were able to inhibit the activating effects of dendritic cells on group 2 innate lymphoid cell, mediated by PGE2 binding on EP2/EP4 (134). TNF-α and IFN-γ were reduced in activated splenocytes, partially through PGE2/COX2 (135). These studies show the importance of bioactive lipids in the therapeutic activity of MSC-EVs.

In the end, multiple mechanism of actions have been proposed, using *in vitro* and *in vivo* models. Taking all these studies into account, the immunomodulatory potential of MSC-EVs is probably mediated not by one specific pathway, but by a combination of all these bioactive molecules. Since these molecules are located in the secretome either as a soluble fraction, in the membrane of the EVs, or inside the EVs, it appears that managing to keep all these bioactive molecules as a part of the secretome downstream process will be key for keeping MSC-related potency.

Many studies discussed before used different techniques to enhance the quantity of immunomodulatory molecules in MSC-EVs. In the next part, we discuss the techniques to obtain MSC-EVs with a higher immunomodulatory potential.

## 4 MSC priming for enhanced immunomodulatory potential

MSCs have an important role in tissue repair and can be mobilized to the sites of tissue damage and inflammation. Thus, MSCs *in vivo* are often subject to inflammatory stimuli such as but not limited to, damage-associated molecular patterns (DAMPs), pathogen-associated molecular patterns (PAMPs), pro-inflammatory cytokines from activated immune cells, or hypoxia (136, 137). Attempts to recreate this environment *in vitro* have been used to increase the immunomodulatory potential of MSC-EVs. As a matter of fact, MSCs have high plasticity regarding their immunomodulatory potential: an anti-inflammatory environment might inhibit the immunosuppressive activity of MSCs, while a pro-inflammatory one will enhance it (138, 139). This can explain why some immunomodulatory molecules such as IDO are only produced under a pro-inflammatory environment. (**Table 1**) summarizes all the priming methods for MSC-EV enhanced immunomodulatory potential.

**Table 1:** MSC-priming for increased MSC derived secretome and EVs enhanced immunomodulatory potential.

Priming used	Cell Type	Bioactive molecule and mean of action	Immunoregulatory effect	Reference
IFN-γ (1000U/mL) TNF-α (1000U/mL) IL-1β (10ng/mL) 24h	Nasal mucosa and BM-MSC EVs	Increase of PD-L1, PD-L2, ICAM-1	Suppressed CD3+ T cells Enhanced therapeutic effects in GvHD mice	(140)
LPS (100ng/mL) 48h	UC-MSC EVs	miR-let-7b Regulation of TLR4/NF-κB/STAT3/AKT signaling pathway	Increased M2 polarization	(141)
LPS	BM-MSC EVs	AKT1/AKT2 signaling	Decreased of pro-inflammatory cytokines levels Promoted M2 polarization Alleviated myocardial injury Reduced post-infarction inflammation	(142)
LPS (10ng to 10μg/mL) Poly (I:C) (100ng to 100μg/mL) 6h	UC-MSC EVs		Decreased of pro-inflammatory cytokines levels Promoted M2 polarization	(143)
LPS (100ng/mL 1ug/mL)	BM-MSC EVs		LPS-concentration dependent variation of macrophages markers	(144)
LPS (1μg/mL) 24h	Periodontal ligament MSC EVs	miR-433-3p TLR2/TLR4/NF-κB p65	Promoted M1 phenotype	(145)
LPS (10ng/mL) Poly (I:C) (1μg/mL) 1h	Human multipotent MSC		Increased expression of immune suppressive factors with LPS Pro inflammatory phenotype with Poly (I:C)	(146)
IFN-γ (10ng/mL) TNF-α (15ng/mL) 72h	Human multipotent MSC EVs	A20 TSG-6	Enhanced T cell suppression Increased levels of immunomodulatory proteins	(147)
IFN-γ (10ng/mL) 48h	Adipose MSC Secretome		Promoted M2 polarization	(148)
IL-1β (25ng/mL) 24h	Human BM-MSC EVs	miR-147b Inhibition of NF-kb pathway	Inhibited inflammatory factors expression in osteoarthritis cells	(149)
IL-1β (10ng/mL) 12h	Mouse MSC EVs	miR-21 targets PDCD4	Higher expression of immunosuppressive factors in MSCs Promoted M2 polarization Alleviated sepsis in mice	(150)
IFN-γ (100ng/mL) 24/48h	UC-MSC EVs		Loss of protection against ischemic acute kidney injury No differences in Treg induction	(151)
TGF-β (10ng/mL) IFN-γ (1000IU/mL) 72h	UC-MSC EVs	IDO, IL-10	Increased proportion of Tregs Higher levels in IDO, IL-10 and IFN-y Similar levels of PBMC proliferation inhibition	(101)
Atorvastatin 1μgmol/L 48h	Mouse MSC EVs	lncRNA H19	Improved cardiac function Ameliorated fibrosis after myocardial infarction Reduced cardiac apoptosis and inflammation	(152)
IDO overexpression	Mouse BM-MSC EVs	IDO	Accelerated repair process after acute kidney injury Reduced fibrosis, inflammation Promoted M2 polarization	(100)
IL-10 overexpression	Human UC-MSC EVs	IL-10	Enhanced suppressive effect on Tcells Differentiation of Th1/Th17 cells Upregulated Tregs Ameliorated autoimmune uveitis	(44)
TSG-6 overexpression	Human BM-MSC EVs	TSG-6	Attenuated scar pathological injury Decreased inflammation	(52)
TGF-β, PTX3, let-7b-5p, miR-21-5p overexpression	Human MSC EVs	TGF-b, PTX3, let-7b-5p, miR-21-5p	Decreased inflammation from Th1 and TH17 cells Suppressed TLR4 and TCR signaling in splenocytes	(25)
CD73 overexpression	Human UC-MSC EVs	CD73, Promotion cAMP/PKA signaling pathway	Decreased inflammation and ATP Promoted M2 polarization Ameliorated recovery after spinal cord injury	(66)
PD-L1 overexpression	Mouse BM-MSC EVs	PD-L1 PD-1/PD-L1 pathway	Alleviated pneumonia Reduced levels of inflammation	(77)
PD-L1 overexpression	BM-MSC EVs	PD-L1	Prolonged allograft survival Increased Treg proportion and suppressive effect on T cell proliferation	(78)
PD-L1 overexpression	Mouse BM-MSC EVs	PD-L1	Inhibited immune cells activation Reduced inflammation in colon Ameliorated ulcerative colitis and psoriasis	(79)
miR-181a overexpression	Human UC-MSC EVs	miR-181a targeting c-Fos gene in PBMCs	Decreased levels of inflammation Increased Treg polarization Retarded ischemic damage *in vivo*	(153)
miR-126 overexpression	Mouse adipose MSC EVs	miR-126	Ameliorated functional recovery after stroke Inhibited microglial activation and inflammation after ischemic stroke	(154)
TRAIL overexpression	Human adult MSC EVs	TRAIL	Induced apoptosis in cancer cell lines	(155)
Hypoxia	Mouse adipose MSC EVs	lncRNA-Gm37494 upregulated	Promoted functional recovery after spinal cord injury Promoted M2 polarization and suppressed inflammation	(156)
Hypoxia	Bone MSC EVs	mIR-216a-5p enrichment TLR4/NF-κB/PI3K/AKT signaling cascades	Promoted M2 polarization Increased functional recovery after spinal cord injury	(157)
IFN-γ (50ng/mL) TNF-α (10ng/mL) IL-1β (10ng/mL) HIF-overexpression	Human dental pulp MSC EVs		Promoted M2 polarization Reduced inflammation and PBMC adhesion Ameliorated fibrosis Attenuated TNBS-induced colitis in mice	(80)
Hypoxia	Human adipose MSC EVs		Improved renal recovery after Ischemic injury Promoted M2 polarization	(158)
Spheroid	Human amnion MSC EVs		Inhibited activated PBMC proliferation	(159)
Spheroid	Human MSC secretome		Aggregation method influenced PGE2 secretion Suppressed T-cell Polarized M2 polarization Enhanced expression of immunomodulatory factors	(160)
Spheroid	Human adipose MSC		Protective effect against colitis Inhibited immune cell infiltration in colon	(161)
Spheroid TNF-α, IFN-γ (20 ng/mL)	Human UC-MSC EVs		Increased HGF levels in secretome Enhanced reduction of NF-kB and pro inflammatory cytokines expression Anti-apoptotic and anti-fibrotic effect	(162)
Spheroid	Human BM-MSC EVs		Lower kynurenine concentration Lower anti-inflammatory effect in lungs and lower anti-fibrotic effect	(163)
Aggregates in WAVE bioreactor	Human BM-MSC EVs		Higher miR-21-5p and miR-22-3p expression Higher inhibition of CD8+ T cell proliferation	(164)
Hollow Fiber 3D culture	Human UC-MSC EVs		Improved renal function after kidney injury Reduced inflammatory factors Repressed T cell proliferation and macrophage infiltration	(165)
Hollow Fiber 3D culture	Human UC-MSC EVs		Decreased expression of inflammatory factors Improved cardiac function in acute myocardial infarction Promoted M2 polarization	(166)

Priming or conditioning MSCs with different methods have shown to induce different EV release, membrane markers, differential uptake and activation of T cell subsets (167). Priming also modifies the miRNA and protein EV cargo (168–170). Importantly, the source of MSCs and the inter-donor variability within the same source of MSCs has an impact on the response to priming and thus the improvement of immunosuppressive effect of MSC-EVs. Peltzer et al. showed that PCA failed to discriminate groups between MSC- EVs from 5 donors without priming, with IFN-γ priming and hypoxia, regarding their differential miRNA expression, showing that inter-individual variability was stronger, especially regarding their response to priming (171). Gorgun et al. showed that while priming had a significant effect on the secretome of MSCs, it did not majorly affect the miRNA in their EVs (172). A recent study was able to discriminate hypoxia and normoxia group looking at their miRNA, possibly underlying a donor disparity between studies (173). Jin et al. showed that priming overcame the MSC inter-donor variability by looking at gene expression (174). Priming with a cocktail of cytokines resulted in two different responses in different donors of MSCs (140). These studies show that priming MSC has significative changes on the biophysical, biochemical and bioactive properties of MSC-EVs, but the changes might be hidden by donor- and source-dependent differences.

One way to recreate the inflammatory conditions of tissue damage is to target Toll-Like Receptors (TLRs), which recognize various types of molecules such as DAMPs and PAMPs. LPS-preconditioned MSC-EVs were more efficient in converting THP-1 to M2 phenotype *in vitro*, and in relieving inflammation *in vivo* than untreated MSC-EVs, notably through miR-let-7b (141). LPS-primed MSC-EVs were significantly more efficient at increasing M2 and reducing M1 polarization *in vitro*, and were more effective in relieving post-infarction inflammation in mice (142). Hwang et al. showed that TLR-3 and 4 primed MSCs secretomes were more successful in reducing pro-inflammatory cytokines from LPS-induced macrophages. EVs were key in increasing the percentage of M2 (143). Notably, Kink et al. showed that the concentration of LPS for priming MSC resulted in EVs having different effects on macrophage receptors expression, which might explain the effect of the next studies (144). Indeed, some studies show that TLR-priming does not always result in enhanced immunosuppressive effect. Recent results from Cui et al. showed that LPS primed MSC-EVs induced a M1 and not a M2 phenotype (145). Previous results from Waterman et al. showed a pro-inflammatory phenotype MSCs after TLR-4 priming, and anti-inflammatory phenotype after TLR-3 priming (146). All in all, these results show that priming through TLRs might be an interesting method to prime MSC-EVs, though some optimization of concentration of priming might be important to achieve the highest enhanced immunosuppressive effect.

A second way to prime MSCs is to use pro-inflammatory cytokines. A study by Cheng et al. showed that priming with IFN-γ and TNF-α resulted with higher suppression of T cell proliferation and induced a different protein profile with higher levels of anti-inflammatory proteins such as TSG-6 and A20 (147). Ragni et al. showed that IFN-γ priming changes the proteins secreted by MSCs and the miRNA content of their EVs, resulting in a diminution of M1 polarization (148). Priming with IL-1β resulted in higher anti-inflammatory activity of EVs, with a significant increase of mir-147b, which partially mediated the immunomodulatory effect of primed MSC-EVs in osteoarthritis cells (149). IL-1β priming on mouse MSC resulted in an enhanced macrophage polarization to M2 *in vivo* and *in vitro*, and a better therapeutic effect on septic mice by MSC-EVs. Equivalent priming also induced higher expression of miR-21, which mediated the effect of MSC-EVs on sepsis (150). Nonetheless, conditioning does not always result in better activity: priming with IFN-γ did not induce a better therapeutic effect in T-cell modulation activity, and induced a loss of protection against ischemic acute kidney injury (151). A few teams have also tried cocktails of different pro-inflammatory molecules, compared to one-molecule priming. A combination of IFN-γ and TGF-β priming resulted in a higher proportion of Treg cells after treatment with EVs, but also elevated levels of IFN-γ, IL-10 and IDO within these EVs. The combination of priming resulted in better immunomodulatory effect than EVs derived from untreated MSC, or MSCs treated with only one of the molecules (101). Hackel et al. showed a variation of response between MSCs when treated with multicytokine combination of IFN-γ, TNF-α and IL-1β. One group of 3 donors secreted more PD-L1 with the full priming compared to two-cytokines priming, and the other group of 3 other donors responded equally with 3 and 2-cytokine priming. Nonetheless, priming still induced higher levels of PD-L1 and PD-L2 compared to non-primed EVs. Higher therapeutic effects of primed MSC-EVs were mediated by PD-1 ligands (140). Priming MSCs with pro-inflammatory components has been shown to modify the whole secretome, elevating the level of anti-inflammatory molecules in the secretome and in EVs, thus mediating higher potency and therapeutical effects of MSC-EVs. Priming with higher number of cytokines seems to induce a higher immunomodulatory effect of EVs.

Other molecules have also been used to prime MSCs. Conditioning with Atorvastatin resulted in a better cardioprotective effect on infarcted rat heart, with a better inhibition of TNF-α and Il-6 in the tissue of the infarct zone (152).

Genetic modifications of MSC producing EVs can improve the immunomodulatory potential of MSC-EVs. A few studies already described above have targeted specific immunomodulatory proteins such as IDO (100), IL-10 (44), TSG-6 (52), TGF-β, PTX3, miR-let7-5p and miR-21-5p (25), CD73 (66), PD-L1 (77–79), in order to secrete more of these specific proteins and induce a better immunoregulatory activity of MSC-EVs. Genetic modifications can also target the expression of bioactive miRNAs. Overexpression of miRNA-181a induced better inhibition of the inflammatory response, increased the percentage of Tregs among PBMCs, and delayed ischemic damage *in vivo* (153). Overexpression of miRNA-126 promoted functional recovery after stroke by suppressing microglia activation (154). Genetic modifications can also target molecules which are not constitutively expressed by MSCs. A study has shown that modified MSCs for the expression of TRAIL secrete EVs with TRAIL, and are able to induce apoptosis in various cell lines (155).

Conditioning of MSC in hypoxia has also been shown to improve the immunomodulatory potential of MSC-EVs. Hypoxic environment leads to increased levels of hypoxia-inducible factors, notably HIF-1a, which regulates many physiological pathways, including angiogenesis (175). The effect of hypoxia on the immune-modulatory properties of BM-MSCs has already been reviewed (176). Extracellular vesicles derived from hypoxia conditioned MSCs were more effective in decreasing levels of pro-inflammatory cytokines and in shifting microglia from M1 to M2 polarization, compared to non-treated MSC-derived EVs (156). Same results were observed by another group (157). Hypoxia was also shown to increase the expression of HGF (172), which we have already discussed the immunomodulatory effect. Gómez-Ferrer et al. showed that double-primed hypoxia + inflammation MSC-derived EVs were more efficient in repolarizing M1 to M2-like phenotype than single-primed inflammation MSC-EVs. These EVs promoted healing in a TNBS induced mouse colitis, partially through reduction of pro-inflammatory cytokines (80). Hypoxic UC-MSC-derived EVs inhibited more efficiently maturation of DCs (177). Hypoxia treated AD-MSC EVs were more efficient in reducing macrophage infiltration, reducing levels of Il-6, though MCP-1 levels were higher, compared to non-treated MSC-EVs, in renal tissue after ischemia reperfusion injury (158). Thus, hypoxia conditioning of MSCs could be a relevant technique to enhance the MSC-EV immunosuppressive functions for chronic diseases.

Finally, a few studies have reported higher potency with EVs derived from MSC cultured in 3D. 3D culture of MSCs englobes a great variety of techniques, including but not limited to microcarriers, diverse scaffolds, microgels, and spheroids, which can be then cultured in bioreactors or other types of vessels. 3D culture better mimics the natural cell conditions, and increases the levels of secreted angiogenic and immunomodulatory factors (178).

Bulati et al. were able to differentially cluster between IFN-γ primed and 3D spheroid cultured MSCs by looking at their miRNA EVs (179). Many studies have shown that MSCs have increased immunomodulatory potential after 3D spheroid culture (159–161). EVs derived from pro-inflammatory primed MSC spheroids had an enhanced anti-inflammatory effect by decreasing the expression of NF-κB, IL-8 and IL-6 in TNF-induced inflammation in HK2 cells, compared to 3D cultured only EVs and 2D cultured EVs (162). Nonetheless, Kusuma et al. have shown contrasting results where 3D spheroid cultured MSC-EVs produced significantly less IDO, and that overall, these EVs had a lower immunosuppressive and therapeutic potency than 2D MSC-EVs (163). Further studies are needed to understand the impact of MSC-EVs derived from spheroids.

Regarding MSC culture in bioreactors, 3D cultured in WAVE bioreactor MSC-EVs induced same decrease in M1 markers expression in macrophages than 2D MSC-EVS, but had enhanced suppression of CD8+ T cell proliferation (164). Hollow Fiber bioreactor systems have gained popularity for the 3D culture of MSC for EV research. 3D cultured MSC-EVs using this system were more efficient than 2D cultured MSC-EVs in alleviating acute kidney injury, notably by reducing inflammatory factors, repressing T cell and macrophage infiltration (165). Sun et al. also used the hollow fiber system, and found that 3D-MSC EVs exhibited a stronger anti-inflammatory effect on stimulated monocytes, but also in acute myocardial infarction rats (166). Thus, 3D culture in bioreactor seems to enhance the immune-modulatory potential of MSC-EVs.

Other than MSCs, other EVs have been showing immunosuppressive potential for a therapeutic approach. Namely, tumor cells like sarcoma cells evade the immune system, which is partially mediated by the release of EVs (180, 181). EVs derived from Ewing Sarcoma induced a pro-inflammatory response on myeloid cells, but impaired the maturation and function of dendritic cells (182). Droste et al. wrote an in-depth review about tumor derived EVs, their effect on immune cells and how *in vivo* animal models help understand the potential of these EVs (183).

Based on their broad immunosuppressive effects and the possibility to enhance them through a variety of techniques, MSC-EVs could be a promising therapeutic solution for the treatment of SLE.

## 5 EVs for diagnosis and treatment of SLE

### 5.1 EVs for diagnosis of SLE

Recent studies suggested that EVs open a new perspective for both diagnosis and treatment of SLE. Several teams reported differences between the EV profile of SLE patients and healthy controls. First, the number of total EV was found to vary. Most studies reported an increase of total EVs in SLE patients (184–189), while Nielsen et al. reported a decrease (190). Apart from the number of EV, their composition has also been shown to differ between SLE patients and healthy controls. Østergaard et al. outlined a decrease in the level of cytoskeletal, mitochondrial and organelle proteins contained in microparticles from SLE patients (191). Additionally, Chuang et al. recently reported an overexpression of Eosinophil Cationic Protein (ECP) in SLE T cell-derived EVs and demonstrated their pro-inflammatory property in a mouse model (192).

Aside from protein-containing EVs, attention was drawn to miRNA-containing EVs. Li et al. reported compared to healthy controls, an increase of miR-21 and miR-155, and a decrease of miR-146a in serum EVs (193). Additionally, the expression of miR-21 and miR-146a were negatively associated with respectively anti-SSA/RO antibodies and anti-dsDNA antibodies, which are important features of SLE pathogenesis. The decrease of miR-146a-containing exosomes in the serum of SLE patients was also demonstrated by Dong et al., who suggested that miR-146a is internalized into MSCs and contributes to MSC senescence in SLE patients by targeting the TRAF6/NF-κB pathway (194). Interestingly, Perez-Hernandez et al. had previously found an increase in the urinary miR-146a-containing exosomes in SLE patients (195), suggesting that the location of the EVs should be also taken into consideration. Furthermore, Tan et al. showed that exosomal miR-451a is downregulated in the serum of SLE patients and correlates with the SLE disease activity and renal damage, due to its implication in intercellular communication (196). It has also been demonstrated that microRNAs-containing exosomes, isolated from the plasma of SLE patients, can activate pDCs through the receptor TLR7 and induce excessive production of IFN-α, leading to a chronic state of inflammation in SLE (197). Overall, the EV profile of SLE patients seem to significantly differ from the one of healthy controls, which outlines them as prominent biomarkers for SLE. Moreover, their presence in various body fluids, such as blood, urine and saliva, guarantee a facilitated access for diagnosis and could replace the rather invasive biopsies traditionally used for monitoring the disease progression.

### 5.2 MSC-EVs for treatment of SLE

Allogenic MSCs have already been used as a potential treatment for SLE (198, 199). As the broad immunosuppressive effect of MSCs is mediated by the secretome and EVs, they represent a potential alternative for the treatment of SLE (200). Multiple studies have already been carried using MRL/lpr mice model. Xie et al. have tested the effect of human umbilical cord MSCs and their EVs on a classical animal model of SLE. They have shown that UC-MSCs exert immunoregulatory effects on SLE, partially mediated by their EVs. UC-MSC-EVs were able to inhibit CD4+ T cells in their model, but lower amounts of TGF-β and IL-17 were found in the supernatant (201). Another study also using UC-MSC-EVs has shown an amelioration of SLE after EV administration in MRL/lpr mice by inducing M2 macrophages polarization and increasing regulatory T cell (202). BM-MSC-EVs promoted anti-inflammatory phenotype of macrophages, and induced recruitment of Tregs in murine lupus nephritis model. Notably, they showed the importance of miR-16 and miR-21 in the polarization of macrophages (203). Another study has compared tooth MSCs and their EVs to treat SLE in the same MRL/lpr mice model. The administration of EVs exerted a therapeutic effect on this model by rescuing the immune microenvironment. Furthermore, they have shown a decreased effect with the presence of RNase, hinting at the importance of RNA in the immunomodulatory potential of EVs (204). BM-MSC derived apoptotic vesicles ameliorated lupus in the same model, by suppressing activated CD4+ T cells (205).

Dou et al. showed that MSC-EVs reduced the expression of pro-inflammatory cytokines and promoted M2 polarization of macrophages, notably through tsRNA-21109. This same RNA is downregulated in SLE patients, which sheds light on possible means of action of MSC-EVs in SLE (206). Chen et al. investigated the effect of MSC-EVs in diffuse alveolar hemorrhage (DAH) mice, an uncommon but fatal complication of SLE. EVs alleviated symptoms of DAH, decreased the expression of pro-inflammatory factors and enhanced M2 polarization (207).

Tu et al. showed a lower expression of miR-19b, an imbalance between Th17 and Tregs, a much higher expression of pro-inflammatory cytokines in PBMCs from SLE patients. UC-MSC EVs treatment increased the expression of miR-19b, regulated the Th17/Tregs balance and reduced the expression of pro-inflammatory factors (208). The amount of B cells in SLE patients is significantly upregulated. UC-MSC-EVs promoted B cell apoptosis, inhibited overactivation and decreased the levels of pro-inflammatory cytokines, possibly through regulation of the upregulated miR-155 in SLE patients (209). Type I IFN release by pDCs is closely related to the severity of SLE. While MSCs have shown to reduce the release of IFN-α and inhibit the function of pDCs (210, 211), no studies regarding the impact of MSC-EVs have been carried. Overall, these studies show the therapeutic potential of MSC-EVs for the treatment of SLE.

## 6 Conclusion

The emerging field of EVs presents a promising avenue for therapeutic treatment. EVs carry a variety of membrane and soluble proteins, and play a key role in immune processes. Thus, EVs could be used for diagnostic as a biomarker, or a therapeutic tool. More specifically, MSC-EVs mediate a broad immunosuppressive effect, showcasing their potential as a cell-free therapy for SLE. Further techniques such as pre-conditioning of MSCs, genetic modification or EV engineering could enhance their immunomodulatory activity and could be applied to further therapeutic applications of EVs.

However, a more specific understanding of whether the immunoregulatory activity is mediated by EV-associated bioactive molecules, soluble factors, or both is needed. The impact of the many sources of heterogeneity in EV studies on these immune mediators should be investigated. The EV field still suffers from barriers such as standardization of isolation and characterization methods, Good Manufacturing Practice (GMP)-compliant large scale production, or specific guidelines for validation of EVs as a therapeutic tool, which in turn hinders the use of EVs for the treatment of SLE. Furthermore, regarding SLE, the role of EVs on pDC activation should be further investigated to understand their potential role on chronic production of IFN-I in SLE. Similarly, a fine characterization of pDC derived EVs content should be carried out prior considering the use of EVs as therapeutic strategy. The effect of MSC-EVs regarding the regulation of interferon production of pDCs should be investigated. Finally, methodological studies on the dosage and administration interval of MSC-EVs in SLE are still essential to advance their therapeutic development.

## Author contributions

CW: Writing – original draft, Writing – review & editing. IS: Writing – original draft, Writing – review & editing. FG: Writing – original draft, Writing – review & editing. J-PH: Writing – original draft, Writing – review & editing. TF: Writing – original draft, Writing – review & editing.
