# Corrigendum: Mesenchymal stromal cell derived extracellular vesicles as a therapeutic tool: immune regulation, MSC priming, and applications to SLE

**DOI:** 10.3389/fimmu.2024.1386412

**Published:** 2024-04-08

**Authors:** Christophe Wong, Ivana Stoilova, Florence Gazeau, Jean-Philippe Herbeuval, Thibaut Fourniols

**Affiliations:** ^1^EVerZom, Paris, France; ^2^Centre National de la Recherche Scientifique (CNRS) Unité Mixte de Recherche (UMR) 8601, Université Paris Cité, Paris, France; ^3^Chemistry and Biology, Modeling and Immunology for Therapy (CBMIT), Université Paris Cité, Paris, France; ^4^Matière et Systèmes Complexes (MSC) UMR CNRS 7057, Université Paris Cité, Paris, France

**Keywords:** extracellular vesicles, secretome, immune regulation, mesenchymal stromal cell, priming, systemic lupus erythematosus

In the published article, there was an error in the Funding statement. A funding information was not included in the article. The correct Funding statement appears below.

